# Redetermination of the crystal structure of di-μ_2_-hydroxido-bis­[di-*tert*-butyl­chlorido­tin(IV)] at 100 K

**DOI:** 10.1107/S2414314623000561

**Published:** 2023-01-26

**Authors:** Hans Reuter

**Affiliations:** a Chemistry, Osnabrück University, Barabarstr. 7, 49069 Osnabrück, Germany; Sunway University, Malaysia

**Keywords:** crystal structure, redetermination, hydrolysis, diorganotin(IV)-hydroxide-halides, hydrogen bonding

## Abstract

The structure of the dimeric title compound, [Sn_2_(C_4_H_9_)_4_Cl_2_(OH)_2_], was redetermined at 100 K by use of an area detector to provide new data to improve the structural parameters for detailed analysis. Noteworthy is the folding of the central, non-symmetric, four-membered [SnO]_2_ ring [dihedral angle about the O⋯O axis = 1.09 (3)°], as well as the elongation of the Sn—Cl bonds [mean value = 2.5096 (4) Å], as a result of inter­molecular O—H⋯Cl hydrogen bonds; the latter lead to a chain-like arrangement of dimeric mol­ecules along [101].

## Abstract 2

The crystal structure of the title compound was redetermined at 100 K in order to achieve improved structural data, especially with respect to the C—C distances and the hydrogen bonding.

## Graphical abstract

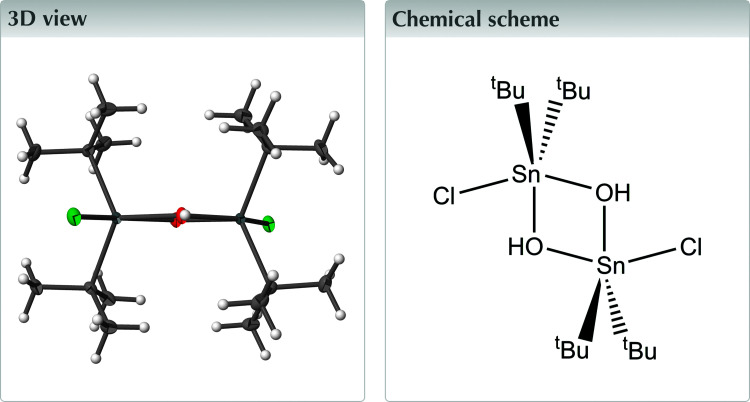

## Structure description

The title compound, [^*t*^Bu_2_Sn(OH)Cl]_2_, belongs to the class of dimeric diorganotin(IV)-hydroxides-halides, [*R*_2_Sn(OH)*X*]_2_, the first hydrolysis products of diorganotin(IV) dihalides, *R*_2_Sn*X*_2_. The structure of the title compound has been determined previously at room temperature using point detector data as part of a paper describing the series of dimeric di-*tert*-butyl­tin(IV) hydroxide halides, [^*t*^Bu_2_Sn(OH)*X*]_2_ with *X* = F, Cl and Br (Puff *et al.*, 1985 ▸). This series was completed when a second modification of the title compound was reported (Di Nicola *et al.*, 2011 ▸) and more recently, when the crystal structures of the pure iodide compound, [^*t*^Bu_2_Sn(OH)I]_2_ (Reuter, 2022 ▸) and its DMSO-adduct (Reuter & Wilberts, 2014 ▸) were published. With two well-resolved, low-temperature crystal-structure determinations of the iodide derivative, it seemed reasonable to redetermine the structure of the chloride derivative using similar experimental conditions to enable a more valid comparison between structures.

**Figure 1:**
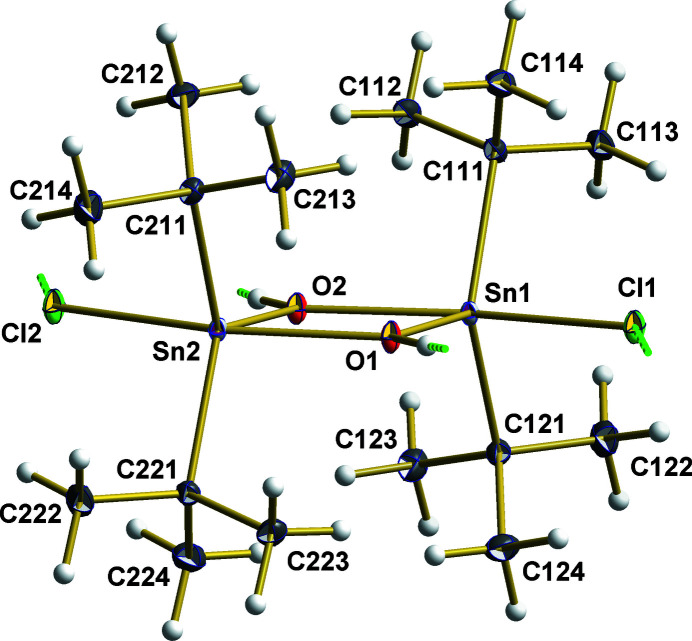
A representation of the dimeric, non-symmetric mol­ecule found in the crystal of [^*t*^Bu_2_Sn(OH)Cl]_2_, showing the atom numbering. With the exception of the hydrogen atoms, which are shown as spheres of arbitrary radius, all other atoms are drawn with displacement ellipsoids at the 40% probability level. Inter­molecular O—H⋯Cl hydrogen bonds are indicated by dashed sticks in green.

As a result of the low-temperature measurement and the high data redundancy, combined with a multi-scan absorption correction, the new data improve the structural parameters of the title compound (Fig. 1 ▸) by an order of magnitude. In particular, the new data enable the confirmation of the exceptionally long Sn—C bonds [range: 2.180 (1) to 2.184 (1) Å; mean value: 2.182 (2) Å] in accord with comparable values found in related [^*t*^Bu_2_Sn(OH)I]_2_ compounds [pure state: *d*(Sn—C)_mean_ = 2.190 (3) Å (Reuter, 2022 ▸), and DMSO-adduct: *d*(Sn—C)_mean_ = 2.193 (10) Å (Reuter & Wilberts, 2014 ▸)].

The other structural features of note relate to the *tert*-butyl groups, which are characterized by C—C bond lengths in the range from 1.521 (2) to 1.532 (2) Å [mean value: 1.527 (4) Å], C_meth­yl_—C—C_meth­yl_ angles in the range 107.1 (1) to 111.1 (1)° [mean value: 109.5 (11)°] and Sn—C—C angles of 107.1 (1) to 111.1 (1)° [mean value: 108.9 (12)°]. These new data are of the same precision and absolute values as those found in the iodide compound both in the pure state [*d*(C—C) = 1.529 (4) Å, 〈(C_meth­yl_—C—C_meth­yl_) = 109.9 (4)°, *d*(Sn—C) = 2.193 (10) Å and 〈(Sn—C—C) = 109.4 (7)° (Reuter, 2022 ▸)], and in the DMSO-adduct [*d*(C—C) = 1.529 (4) Å, 〈(C_meth­yl_—C—C_meth­yl_) = 109.9 (4)° and 〈(Sn—C—C) = 109.4 (7)° (Reuter & Wilberts, 2014 ▸)].

**Figure 2:**
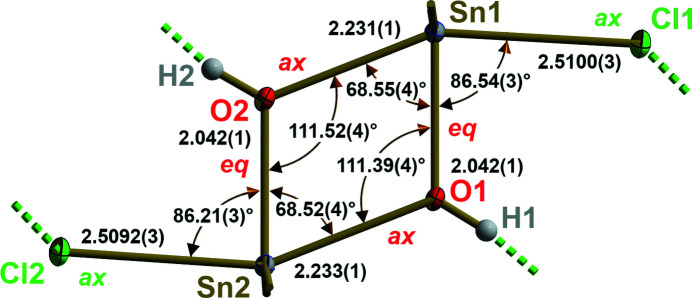
Ball-and-stick model of the inorganic framework of the [^*t*^Bu_2_Sn(OH)Cl]_2_ mol­ecule highlighting selected bond lengths (Å) and angles (°). Positions of oxygen and chloride atoms within the trigonal–biypramidal coordination of the tin atoms are labelled by use of the abbreviation *ax* (= *axial*) and *eq* (= *equatorial*). For clarity, ^*t*^Bu groups are stripped down to the Sn—C bonds drawn as shortened sticks. Inter­molecular O—H⋯Cl hydrogen bonds are indicated by dashed sticks in green.

As the mol­ecule belongs to point group *C*_1_, the central, four-membered, rhombic [SnO]_2_ ring is not exactly planar but folded along the O⋯O axis with an inter­planar angle of 1.09 (3)°. As usual, the bond lengths and angles within the inorganic part of the mol­ecule (Fig. 2 ▸) are characteristic of tin(IV) in trigonal–bipyramidal coordination (*ax*/*eq*), and the size of the *tert*-butyl groups. A special feature of the title compound relates to the Sn—Cl distances [mean value: 2.5096 (4) Å], which are considerably longer in comparison with other Brønstedt-Base (BB) stabilized diorganotin(IV)-hydroxide-chlorides [*R* = Ph, BB = EtOH: *d*(Sn—Cl) = 2.4748 (6) Å (Barba *et al.*, 2007 ▸); *R* = Ph, BB = quinoline: *d*(Sn—Cl) = 2.4648 (11)/2.4353 (12) Å (Anacona *et al.*, 2003 ▸)].

**Figure 3:**
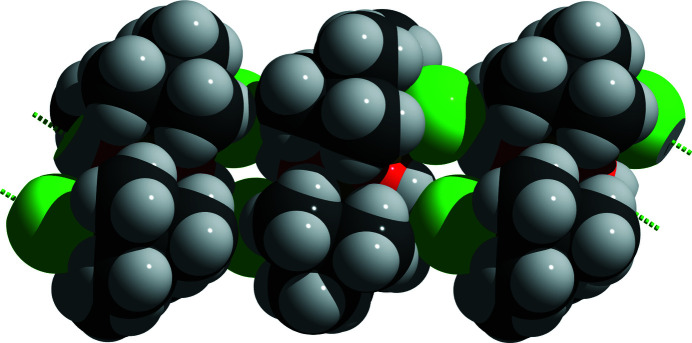
Space-filling model of the chain-like arrangement of the [^*t*^Bu_2_Sn(OH)Cl)]_2_ mol­ecules along [101] encompassing intra­molecular O—H⋯Cl hydrogen bonding. The image shows three complete mol­ecules with their hydrogen bonds to neighbouring mol­ecules. The corresponding atoms are visualized as truncated, two-coloured spheres and the hydrogen bonds are indicated by dashed sticks in green. Colour code of the atoms: Cl = green, H = white, C = grey, O = red, Sn = brass.

These unusually long Sn—Cl bonds in the title compound arise from the fact that the chloride atoms are involved in inter­molecular O—H⋯Cl hydrogen bonds (Table 1 ▸), resulting in a chain-like arrangement of the [^*t*^Bu_2_Sn(OH)Cl]_2_ mol­ecules along [101], Fig. 3 ▸, a feature that had been recognized previously but now is confirmed unambiguously.

## Synthesis and crystallization

For the synthesis of the title compound, see Puff *et al.* (1985 ▸).

## Refinement

Crystal data, data collection and structure refinement details are summarized in Table 2 ▸. Six reflections were omitted from the final cycles of refinement owing to poor agreement; details are given in the CIF.

## Supplementary Material

Crystal structure: contains datablock(s) I. DOI: 10.1107/S2414314623000561/tk4087sup1.cif

Structure factors: contains datablock(s) I. DOI: 10.1107/S2414314623000561/tk4087Isup2.hkl

CCDC reference: 2237568

Additional supporting information:  crystallographic information; 3D view; checkCIF report

## Figures and Tables

**Table 1:** Hydrogen-bond geometry (Å, °)

*D*—H⋯*A*	*D*—H	H⋯*A*	*D*⋯*A*	*D*—H⋯*A*
O1—H1⋯Cl2^i^	0.96	2.30	3.2469 (10)	169
O2—H2⋯Cl1^ii^	0.96	2.31	3.2482 (10)	166

Symmetry codes: (i) 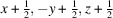 ; (ii) 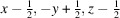 .

**Table 2:** Experimental details

Crystal data	
Chemical formula	[Sn_2_(C_4_H_9_)_4_Cl_2_(OH)_2_]
*M*_r_	570.74
Crystal system, space group	Monoclinic, *P*2_1_/*n*
Temperature (K)	100
*a*, *b*, *c* (Å)	11.0632 (3), 16.9135 (5), 13.4178 (3)
β (°)	110.442 (1)
*V* (Å^3^)	2352.60 (11)
*Z*	4
Radiation type	Mo *K*α
μ (mm^−1^)	2.36
Crystal size (mm)	0.17 × 0.09 × 0.07

Data collection	
Diffractometer	Bruker APEXII CCD
Absorption correction	Multi-scan (*SADABS*; Krause *et al.*, 2015 ▸)
*T*_min_, *T*_max_	0.515, 0.745
No. of measured, independent and observed [*I* > 2σ(*I*)] reflections	114387, 5668, 5148
*R*_int_	0.031
(sin θ/λ)_max_ (Å^−1^)	0.660

Refinement	
*R*[*F* ^2^ > 2σ(*F* ^2^)], *wR*(*F* ^2^), *S*	0.014, 0.034, 1.06
No. of reflections	5668
No. of parameters	224
H-atom treatment	Only H-atom displacement parameters refined
Δρ_max_, Δρ_min_ (e Å^−3^)	0.94, −0.49

Computer programs: *APEX2* and *SAINT* (Bruker, 2009 ▸), *SHELXS97* (Sheldrick, 2008 ▸), *SHELXL2014/7* (Sheldrick, 2015 ▸), *DIAMOND* (Brandenburg, 2006 ▸), *Mercury* (Macrae *et al.*, 2020 ▸) and *publCIF* (Westrip, 2010 ▸).

## full crystallographic data

### Crystal data

**Table:**

[Sn_2_(C_4_H_9_)_4_Cl_2_(OH)_2_]	*F*(000) = 1136
*M**_r_* = 570.74	*D*_x_ = 1.611 Mg m^−^^3^
Monoclinic, *P*2_1_/*n*	Mo *K*α radiation, λ = 0.71073 Å
*a* = 11.0632 (3) Å	Cell parameters from 9559 reflections
*b* = 16.9135 (5) Å	θ = 3.0–28.0°
*c* = 13.4178 (3) Å	µ = 2.36 mm^−^^1^
β = 110.442 (1)°	*T* = 100 K
*V* = 2352.60 (11) Å^3^	Bloc, colourless
*Z* = 4	0.17 × 0.09 × 0.07 mm

### Data collection

**Table:**

Bruker APEXII CCD diffractometer	5148 reflections with *I* > 2σ(*I*)
φ and ω scans	*R*_int_ = 0.031
Absorption correction: multi-scan (SADABS; Krause *et al.*, 2015)	θ_max_ = 28.0°, θ_min_ = 3.1°
*T*_min_ = 0.515, *T*_max_ = 0.745	*h* = −14→14
114387 measured reflections	*k* = −22→22
5668 independent reflections	*l* = −17→17

### Refinement

**Table:**

Refinement on *F*^2^	0 restraints
Least-squares matrix: full	Hydrogen site location: mixed
*R*[*F*^2^ > 2σ(*F*^2^)] = 0.014	Only H-atom displacement parameters refined
*wR*(*F*^2^) = 0.034	*w* = 1/[σ^2^(*F*_o_^2^) + (0.0158*P*)^2^ + 1.0738*P*] where *P* = (*F*_o_^2^ + 2*F*_c_^2^)/3
*S* = 1.06	(Δ/σ)_max_ = 0.002
5668 reflections	Δρ_max_ = 0.94 e Å^−^^3^
224 parameters	Δρ_min_ = −0.49 e Å^−^^3^

### Special details

**Table:**

Geometry. All esds (except the esd in the dihedral angle between two l.s. planes) are estimated using the full covariance matrix. The cell esds are taken into account individually in the estimation of esds in distances, angles and torsion angles; correlations between esds in cell parameters are only used when they are defined by crystal symmetry. An approximate (isotropic) treatment of cell esds is used for estimating esds involving l.s. planes.
Refinement. Six reflections, i.e. (-1 1 1), (1 1 0), (0 2 1), (0 1 1), (1 0 1) and (0 0 2), were omitted from the final cycles of refinement owing to poor agreement.The positions of all H atoms were clearly identified in difference Fourier syntheses. Those of the *tert*-butyl groups were refined with calculated positions (C—H = 0.98 Å) and common *U*_iso_(H) parameters for each of the methyl groups. The position of the H atoms of the OH groups were refined with a fixed O—H distance of 0.96 Å before they were fixed and allowed to ride on their parent atoms with a common *U*_iso_(H) parameter.

### Fractional atomic coordinates and isotropic or equivalent isotropic displacement parameters (Å^2^)

**Table:**

	*x*	*y*	*z*	*U*_iso_*/*U*_eq_	
Sn1	0.73501 (2)	0.26288 (2)	0.64133 (2)	0.00989 (3)	
Sn2	0.42676 (2)	0.22975 (2)	0.65688 (2)	0.01001 (3)	
Cl1	0.92792 (3)	0.29807 (2)	0.80086 (3)	0.01622 (7)	
Cl2	0.23245 (3)	0.20159 (2)	0.49523 (3)	0.01753 (7)	
O1	0.63487 (9)	0.25652 (6)	0.74333 (7)	0.01351 (19)	
H1	0.6748	0.2674	0.8179	0.039 (4)*	
O2	0.52774 (9)	0.23420 (6)	0.55544 (8)	0.0136 (2)	
H2	0.4839	0.2239	0.4812	0.039 (4)*	
C111	0.72855 (13)	0.37640 (8)	0.56249 (11)	0.0143 (3)	
C112	0.61905 (14)	0.37644 (9)	0.45494 (11)	0.0203 (3)	
H11A	0.6206	0.4260	0.4177	0.025 (3)*	
H11B	0.5363	0.3715	0.4658	0.025 (3)*	
H11C	0.6299	0.3318	0.4123	0.025 (3)*	
C113	0.85829 (14)	0.38736 (9)	0.54696 (12)	0.0201 (3)	
H11D	0.8704	0.3449	0.5017	0.027 (3)*	
H11E	0.9281	0.3857	0.6162	0.027 (3)*	
H11F	0.8593	0.4385	0.5130	0.027 (3)*	
C114	0.70965 (15)	0.44211 (9)	0.63384 (12)	0.0202 (3)	
H11G	0.7103	0.4935	0.6004	0.033 (3)*	
H11H	0.7797	0.4402	0.7030	0.033 (3)*	
H11I	0.6268	0.4347	0.6439	0.033 (3)*	
C121	0.81282 (13)	0.15162 (8)	0.60755 (11)	0.0149 (3)	
C122	0.94120 (15)	0.16949 (10)	0.59347 (14)	0.0267 (4)	
H12A	0.9778	0.1204	0.5774	0.025 (3)*	
H12B	1.0011	0.1925	0.6592	0.025 (3)*	
H12C	0.9271	0.2070	0.5348	0.025 (3)*	
C123	0.71987 (15)	0.11464 (9)	0.50566 (12)	0.0217 (3)	
H12D	0.7040	0.1520	0.4467	0.030 (3)*	
H12E	0.6382	0.1021	0.5153	0.030 (3)*	
H12F	0.7578	0.0661	0.4896	0.030 (3)*	
C124	0.83528 (16)	0.09618 (9)	0.70159 (13)	0.0252 (3)	
H12G	0.7532	0.0860	0.7118	0.034 (3)*	
H12H	0.8958	0.1206	0.7659	0.034 (3)*	
H12I	0.8713	0.0462	0.6877	0.034 (3)*	
C211	0.35215 (13)	0.34122 (8)	0.69386 (11)	0.0148 (3)	
C212	0.32310 (15)	0.39629 (9)	0.59874 (12)	0.0224 (3)	
H21A	0.2627	0.3704	0.5355	0.031 (3)*	
H21B	0.4033	0.4087	0.5865	0.031 (3)*	
H21C	0.2845	0.4452	0.6129	0.031 (3)*	
C213	0.44854 (15)	0.37962 (9)	0.79313 (12)	0.0216 (3)	
H21D	0.5282	0.3922	0.7801	0.027 (3)*	
H21E	0.4679	0.3430	0.8533	0.027 (3)*	
H21F	0.4113	0.4283	0.8097	0.027 (3)*	
C214	0.22696 (15)	0.32214 (10)	0.71335 (14)	0.0248 (3)	
H21G	0.1663	0.2964	0.6502	0.036 (3)*	
H21H	0.1888	0.3712	0.7277	0.036 (3)*	
H21I	0.2457	0.2866	0.7746	0.036 (3)*	
C221	0.42891 (13)	0.11423 (8)	0.72947 (11)	0.0153 (3)	
C222	0.29620 (15)	0.10024 (10)	0.73817 (13)	0.0255 (4)	
H22A	0.2296	0.1034	0.6673	0.037 (3)*	
H22B	0.2799	0.1406	0.7843	0.037 (3)*	
H22C	0.2942	0.0478	0.7685	0.037 (3)*	
C223	0.53316 (14)	0.11266 (9)	0.84006 (11)	0.0199 (3)	
H22D	0.6181	0.1192	0.8335	0.025 (3)*	
H22E	0.5301	0.0620	0.8744	0.025 (3)*	
H22F	0.5181	0.1558	0.8831	0.025 (3)*	
C224	0.45453 (16)	0.05138 (9)	0.65775 (13)	0.0241 (3)	
H22G	0.3885	0.0548	0.5867	0.031 (3)*	
H22H	0.4518	−0.0011	0.6877	0.031 (3)*	
H22I	0.5398	0.0601	0.6528	0.031 (3)*	

### Atomic displacement parameters (Å^2^)

**Table:**

	*U*^11^	*U*^22^	*U*^33^	*U*^12^	*U*^13^	*U*^23^
Sn1	0.00886 (5)	0.01220 (5)	0.00797 (5)	−0.00117 (3)	0.00212 (3)	0.00024 (3)
Sn2	0.00854 (5)	0.01264 (5)	0.00809 (5)	−0.00064 (3)	0.00195 (3)	0.00097 (3)
Cl1	0.01183 (14)	0.02417 (18)	0.00977 (14)	−0.00356 (13)	0.00015 (12)	0.00008 (13)
Cl2	0.01190 (14)	0.02750 (19)	0.00997 (15)	−0.00395 (13)	−0.00023 (12)	0.00079 (13)
O1	0.0099 (4)	0.0218 (5)	0.0079 (4)	−0.0028 (4)	0.0021 (4)	−0.0015 (4)
O2	0.0107 (4)	0.0209 (5)	0.0085 (4)	−0.0024 (4)	0.0025 (4)	−0.0011 (4)
C111	0.0147 (6)	0.0146 (7)	0.0125 (6)	−0.0035 (5)	0.0031 (5)	0.0011 (5)
C112	0.0209 (7)	0.0215 (8)	0.0138 (7)	−0.0043 (6)	0.0000 (6)	0.0042 (6)
C113	0.0190 (7)	0.0229 (8)	0.0198 (7)	−0.0036 (6)	0.0087 (6)	0.0044 (6)
C114	0.0228 (7)	0.0156 (7)	0.0213 (7)	−0.0016 (6)	0.0063 (6)	−0.0013 (6)
C121	0.0125 (6)	0.0151 (7)	0.0159 (7)	0.0001 (5)	0.0034 (5)	−0.0020 (5)
C122	0.0184 (7)	0.0250 (8)	0.0406 (10)	−0.0003 (6)	0.0153 (7)	−0.0069 (7)
C123	0.0201 (7)	0.0223 (8)	0.0199 (7)	0.0015 (6)	0.0036 (6)	−0.0080 (6)
C124	0.0274 (8)	0.0190 (8)	0.0243 (8)	0.0044 (6)	0.0030 (7)	0.0037 (6)
C211	0.0132 (6)	0.0162 (7)	0.0147 (7)	0.0016 (5)	0.0042 (5)	−0.0001 (5)
C212	0.0239 (8)	0.0178 (7)	0.0229 (8)	0.0038 (6)	0.0047 (6)	0.0042 (6)
C213	0.0192 (7)	0.0228 (8)	0.0197 (7)	0.0031 (6)	0.0028 (6)	−0.0066 (6)
C214	0.0189 (7)	0.0253 (8)	0.0343 (9)	0.0009 (6)	0.0143 (7)	−0.0034 (7)
C221	0.0134 (6)	0.0156 (7)	0.0156 (7)	−0.0016 (5)	0.0037 (5)	0.0036 (5)
C222	0.0182 (7)	0.0295 (9)	0.0288 (9)	−0.0037 (6)	0.0082 (7)	0.0122 (7)
C223	0.0190 (7)	0.0218 (8)	0.0163 (7)	0.0012 (6)	0.0029 (6)	0.0070 (6)
C224	0.0277 (8)	0.0172 (7)	0.0247 (8)	−0.0019 (6)	0.0059 (7)	−0.0017 (6)

### Geometric parameters (Å, º)

**Table:**

Sn1—O1	2.0424 (10)	C123—H12D	0.9800
Sn1—C111	2.1813 (14)	C123—H12E	0.9800
Sn1—C121	2.1818 (14)	C123—H12F	0.9800
Sn1—O2	2.2306 (9)	C124—H12G	0.9800
Sn1—Cl1	2.5100 (3)	C124—H12H	0.9800
Sn2—O2	2.0416 (10)	C124—H12I	0.9800
Sn2—C221	2.1796 (14)	C211—C212	1.521 (2)
Sn2—C211	2.1836 (14)	C211—C213	1.5301 (19)
Sn2—O1	2.2329 (9)	C211—C214	1.531 (2)
Sn2—Cl2	2.5092 (3)	C212—H21A	0.9800
O1—H1	0.9600	C212—H21B	0.9800
O2—H2	0.9600	C212—H21C	0.9800
C111—C112	1.5261 (19)	C213—H21D	0.9800
C111—C114	1.528 (2)	C213—H21E	0.9800
C111—C113	1.532 (2)	C213—H21F	0.9800
C112—H11A	0.9800	C214—H21G	0.9800
C112—H11B	0.9800	C214—H21H	0.9800
C112—H11C	0.9800	C214—H21I	0.9800
C113—H11D	0.9800	C221—C224	1.525 (2)
C113—H11E	0.9800	C221—C223	1.5289 (19)
C113—H11F	0.9800	C221—C222	1.531 (2)
C114—H11G	0.9800	C222—H22A	0.9800
C114—H11H	0.9800	C222—H22B	0.9800
C114—H11I	0.9800	C222—H22C	0.9800
C121—C124	1.521 (2)	C223—H22D	0.9800
C121—C122	1.527 (2)	C223—H22E	0.9800
C121—C123	1.5283 (19)	C223—H22F	0.9800
C122—H12A	0.9800	C224—H22G	0.9800
C122—H12B	0.9800	C224—H22H	0.9800
C122—H12C	0.9800	C224—H22I	0.9800
			
O1—Sn1—C111	115.96 (5)	H12B—C122—H12C	109.5
O1—Sn1—C121	116.07 (5)	C121—C123—H12D	109.5
C111—Sn1—C121	127.37 (5)	C121—C123—H12E	109.5
O1—Sn1—O2	68.55 (4)	H12D—C123—H12E	109.5
C111—Sn1—O2	95.00 (4)	C121—C123—H12F	109.5
C121—Sn1—O2	96.55 (4)	H12D—C123—H12F	109.5
O1—Sn1—Cl1	86.54 (3)	H12E—C123—H12F	109.5
C111—Sn1—Cl1	94.53 (4)	C121—C124—H12G	109.5
C121—Sn1—Cl1	95.86 (4)	C121—C124—H12H	109.5
O2—Sn1—Cl1	155.04 (3)	H12G—C124—H12H	109.5
O2—Sn2—C221	114.07 (5)	C121—C124—H12I	109.5
O2—Sn2—C211	117.25 (5)	H12G—C124—H12I	109.5
C221—Sn2—C211	128.20 (5)	H12H—C124—H12I	109.5
O2—Sn2—O1	68.52 (4)	C212—C211—C213	110.44 (12)
C221—Sn2—O1	95.82 (4)	C212—C211—C214	109.50 (12)
C211—Sn2—O1	96.31 (4)	C213—C211—C214	109.74 (12)
O2—Sn2—Cl2	86.21 (3)	C212—C211—Sn2	108.90 (10)
C221—Sn2—Cl2	95.07 (4)	C213—C211—Sn2	111.13 (9)
C211—Sn2—Cl2	94.74 (4)	C214—C211—Sn2	107.05 (10)
O1—Sn2—Cl2	154.72 (3)	C211—C212—H21A	109.5
Sn1—O1—Sn2	111.39 (4)	C211—C212—H21B	109.5
Sn1—O1—H1	121.9	H21A—C212—H21B	109.5
Sn2—O1—H1	126.6	C211—C212—H21C	109.5
Sn2—O2—Sn1	111.52 (4)	H21A—C212—H21C	109.5
Sn2—O2—H2	119.3	H21B—C212—H21C	109.5
Sn1—O2—H2	129.2	C211—C213—H21D	109.5
C112—C111—C114	110.79 (12)	C211—C213—H21E	109.5
C112—C111—C113	110.09 (12)	H21D—C213—H21E	109.5
C114—C111—C113	109.69 (12)	C211—C213—H21F	109.5
C112—C111—Sn1	109.86 (9)	H21D—C213—H21F	109.5
C114—C111—Sn1	108.95 (9)	H21E—C213—H21F	109.5
C113—C111—Sn1	107.39 (9)	C211—C214—H21G	109.5
C111—C112—H11A	109.5	C211—C214—H21H	109.5
C111—C112—H11B	109.5	H21G—C214—H21H	109.5
H11A—C112—H11B	109.5	C211—C214—H21I	109.5
C111—C112—H11C	109.5	H21G—C214—H21I	109.5
H11A—C112—H11C	109.5	H21H—C214—H21I	109.5
H11B—C112—H11C	109.5	C224—C221—C223	110.91 (12)
C111—C113—H11D	109.5	C224—C221—C222	109.56 (13)
C111—C113—H11E	109.5	C223—C221—C222	109.98 (12)
H11D—C113—H11E	109.5	C224—C221—Sn2	108.55 (10)
C111—C113—H11F	109.5	C223—C221—Sn2	109.63 (9)
H11D—C113—H11F	109.5	C222—C221—Sn2	108.15 (10)
H11E—C113—H11F	109.5	C221—C222—H22A	109.5
C111—C114—H11G	109.5	C221—C222—H22B	109.5
C111—C114—H11H	109.5	H22A—C222—H22B	109.5
H11G—C114—H11H	109.5	C221—C222—H22C	109.5
C111—C114—H11I	109.5	H22A—C222—H22C	109.5
H11G—C114—H11I	109.5	H22B—C222—H22C	109.5
H11H—C114—H11I	109.5	C221—C223—H22D	109.5
C124—C121—C122	109.58 (13)	C221—C223—H22E	109.5
C124—C121—C123	110.72 (12)	H22D—C223—H22E	109.5
C122—C121—C123	109.55 (13)	C221—C223—H22F	109.5
C124—C121—Sn1	108.74 (10)	H22D—C223—H22F	109.5
C122—C121—Sn1	107.84 (10)	H22E—C223—H22F	109.5
C123—C121—Sn1	110.35 (9)	C221—C224—H22G	109.5
C121—C122—H12A	109.5	C221—C224—H22H	109.5
C121—C122—H12B	109.5	H22G—C224—H22H	109.5
H12A—C122—H12B	109.5	C221—C224—H22I	109.5
C121—C122—H12C	109.5	H22G—C224—H22I	109.5
H12A—C122—H12C	109.5	H22H—C224—H22I	109.5

### Hydrogen-bond geometry (Å, º)

**Table:**

*D*—H···*A*	*D*—H	H···*A*	*D*···*A*	*D*—H···*A*
O1—H1···Cl2^i^	0.96	2.30	3.2469 (10)	169
O2—H2···Cl1^ii^	0.96	2.31	3.2482 (10)	166

Symmetry codes: (i) *x*+1/2, −*y*+1/2, *z*+1/2; (ii) *x*−1/2, −*y*+1/2, *z*−1/2.
